# New and emerging pharmacologic treatments for MDD

**DOI:** 10.3389/fpsyt.2025.1621887

**Published:** 2025-08-08

**Authors:** Aslihan Uyar, Ali Saffet Gonul

**Affiliations:** ^1^ Standardization of Computational Anatomy Techniques for Cognitive and Behavioral Sciences (SoCAT) Lab, Department of Psychiatry, Faculty of the Medicine, Ege University, Izmir, Türkiye; ^2^ Department of Psychiatry, Mugla Sıtkı Kocman University Training and Research Hospital, Mugla, Türkiye

**Keywords:** psychopharmacology, treatment resistant depression, neuroplasticity, pharmacologic innovations, rapid-acting therapies

## Abstract

Major depressive disorder (MDD) presents a significant global health challenge, characterized by a high prevalence and significant impact on quality of life. Traditional antidepressants fall short in terms of efficacy and onset speed, up to 60% of patients. This review delves into the new and emerging pharmacologic treatments for MDD, focusing on their mechanisms of action, clinical effectiveness, and potential to fill the gaps left by conventional therapies. New and emerging treatments in MDD have centered on different neurobiological pathways than the traditional monoaminergic systems. Ketamine and its enantiomer, S-ketamine, have been highlighted for their rapid antidepressant effects, which act through non-competitive *N*-methyl-d-aspartate (NMDA) receptor antagonism and other pathways involving synaptic plasticity. Clinical trials have demonstrated the ability of ketamine to quickly reduce symptoms, particularly in treatment-resistant cases, with effects noticeable within hours and lasting several days post-administration. Furthermore, the combination of dextromethorphan and bupropion has shown promise. This formulation leverages the NMDA receptor antagonism and sigma-1 receptor agonism of dextromethorphan, complemented by the inhibition of monoamine uptake and metabolism by bupropion, resulting in quicker and more durable antidepressant effects compared with monotherapy. Neurosteroids such as brexanolone and zuranolone, which target γ-aminobutyric acid (GABA)-A receptors, have emerged as effective treatments for postpartum depression. Brexanolone, administered via infusion, and zuranolone, available as an oral formulation, both have demonstrated efficacy in clinical settings. Novel treatments targeting opioid pathways, such as esmethadone, and selective kappa receptor antagonists offer new hope for addressing the symptoms of MDD through mechanisms not traditionally associated with antidepressant action.

## Introduction

Major depressive disorder (MDD) is one of the most common psychiatric disorders and the leading cause of disability worldwide. The global prevalence of this frequently recurrent disease is 4.4%, while its lifetime prevalence is around 16% (52). In many patients, the first episode of MDD manifests in mid–late adolescence and can be unrecognized for up to 8 years (1). The median 1-year prevalence in this age group is similar to that in adult groups (1). Unfortunately, the prevalence of depression increases in early adulthood, while that in the other age groups remains steady. This brings the issue of a larger depressed population in the near future. On the other hand, despite the large armamentarium of treatment options, the goal of reaching remission for patients is still low and does not exceed 40% (2). The fact that the effect size between placebo and standardized drug in controlled clinical trials is only 0.3 suggests that there is an overlap in up to 88% of the depression scores of the two groups and that only 62% of depressed patients on active drugs have lower scores than those on placebo at the end of the trial (3). In the STAR*D study, After the first antidepressant, 67% of patients can achieve remission after a four-step intervention of augmentation and a combination of treatments (4). It is noticeable that the rate of remission at the end of each sequential treatment decreased. Recent revaluation of the STAR*D data proposed that the previously obtained numbers might be lower for remission rates, suggesting that reaching remission might be harder than previously reported in STAR*D (5). Furthermore, persistent symptoms in the partial responders or suboptimally treated patients, such as anhedonia, sleep disorders, and cognitive impairment, increase both the burden of disease and the risk of relapse (4, 6). Nearly 60% of patients with MDD discontinue their antidepressant medication due to side effects, lack of efficacy, and fear of being stigmatized or becoming addicted to them. Indeed, side effects such as sexual dysfunction, weight gain, and sleep disturbances might be persistent, while most gastrointestinal side effects are temporary. Therefore, nearly half of the patients who achieved remission during the acute treatment period (12 weeks) cannot stay in remission in the next 12 months (7). One other problem with the current widely available antidepressants is their late-onset action (2–12 weeks) and low functional recovery (8). Only a group of patients in remission can return to full functional recovery.

**Figure 1:**
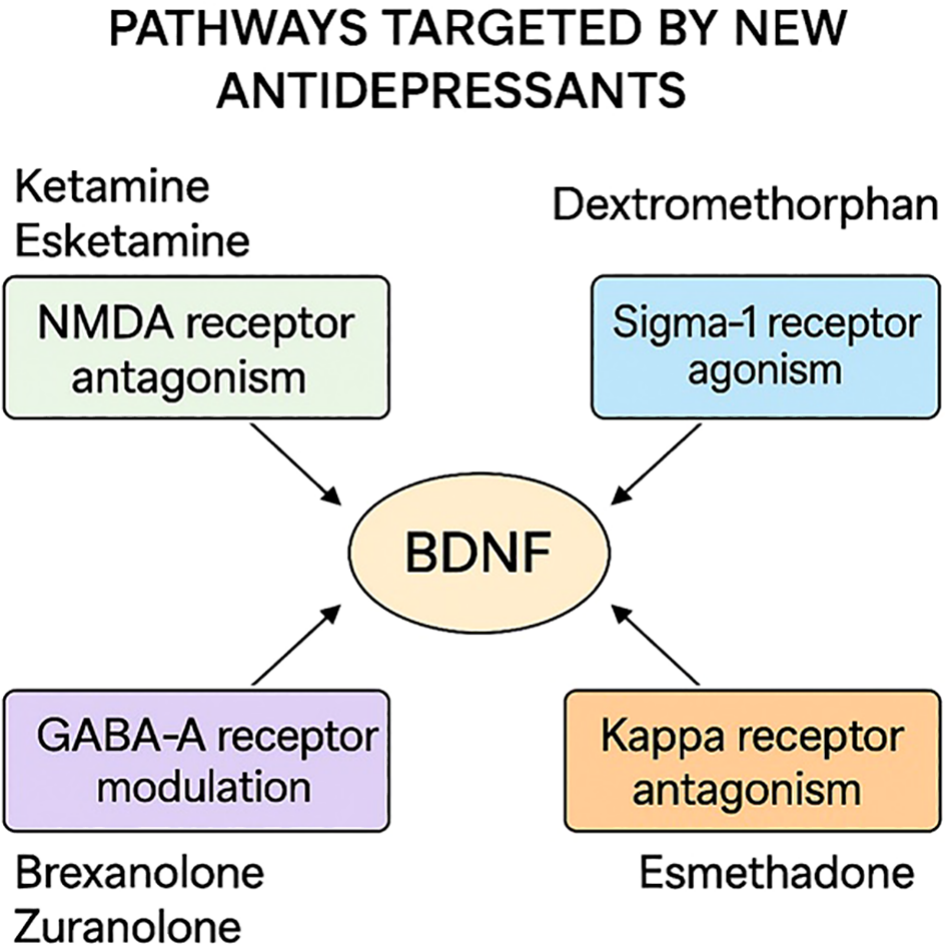
Mechanisms of action of novel antidepressants discussed in this review, including NMDA receptor modulation, GABA-A receptor potentiation, and multimodal serotonergic activity.

Most of the currently available conventional antidepressants exert their effects on similar monoaminergic neurotransmitters such as serotonin (5-HT) or norepinephrine. The differences between them lie in the occupancy of other receptors or pharmacokinetic specialties, which may be related to side or adjuvant effects. Indeed, their comparison showed no or very little efficacy differences (6). Therefore, there is a need for new therapetic agents that are better tolerable, fast-acting, and possibly effective on other neurological systems than monoamine antidepressants. Antidepressants that are effective on other peptides or receptors might also have an advantage in combining with conventional monoaminergic antidepressants in treatment-resistant patients who are currently defined as non-responsive to trials of antidepressants (**Figure 1**).

In this narrative review, we aimed to comprehensively assess new and emerging pharmacological therapies for MDD, focusing on their mechanisms, regulatory status, and clinical evidence from recent trials. We will emphasize the mechanisms of action of these treatments and attempt to offer a framework for understanding their clinical utility.

## Review methodology

This article is a narrative review based on the authors’ synthesis of recent literature published between 2018 and 2025. The selection of treatments and studies was informed by clinical relevance, novelty of the mechanism, and regulatory significance. No systematic search or PRISMA methodology was employed. **Table 1** summarizes the key features, mechanisms, and regulatory statuses of the novel antidepressants discussed in this review.

**Table 1:** Summary of novel and emerging pharmacologic agents for major depressive disorder (MDD).

Agent	Mechanism of action	Route of administration	Indication	Experimental evidence	FDA status	Year introduced	Notable adverse effects
Esketamine	NMDA receptor antagonist; increases glutamate signaling	Intranasal	Treatment-resistant depression (TRD)	Multiple RCTs (e.g., TRANSFORM, SUSTAIN trials); real-world data (e.g., REAL-ESK study)	Approved for TRD (2019)	2019	Dissociation, blood pressure elevation, abuse potential, sedation
Zuranolone	Positive allosteric modulator of GABA_A receptor	Oral	Postpartum depression, MDD	Phase 3 trials (e.g., MOUNTAIN, WATERFALL studies) showed efficacy over placebo	Approved for PPD (2023); MDD under review	2023	Somnolence, dizziness, sedation
Esmethadone (REL-1017)	Uncompetitive NMDA receptor antagonist	Oral	MDD (adjunctive or monotherapy)	Phase 2 and 3 trials demonstrated rapid antidepressant effects without dissociation or psychotomimetic effects	Not yet approved	–	Mild dizziness, headache, well-tolerated profile
AXS-05 (Dextromethorphan–bupropion)	Sigma-1 receptor agonist and NMDA receptor antagonist (dextromethorphan); dopamine/norepinephrine reuptake inhibitor (bupropion)	Oral	MDD	Strong efficacy in the GEMINI and ASCEND trials; comparative advantages in onset of action and symptom domains	Approved (2022)	2022	Dizziness, dry mouth, insomnia
Psilocybin	5-HT2A receptor partial agonist; modulates default mode network (DMN) activity	Oral (single dose, supervised setting)	MDD, TRD, existential anxiety	Phase 2 RCTs (e.g., COMPASS trial); meta-analyses support sustained effects; neuroimaging studies show DMN modulation	Not FDA-approved; Breakthrough status	–	Hallucinations, emotional lability, anxiety during session
SAGE-217 (Zuranolone)	GABA_A receptor positive allosteric modulator	Oral	PPD, MDD	Similar to zuranolone profile; fast-acting with good tolerability	Same as above	–	See zuranolone
Navacaprant (MK-1942)	Selective kappa-opioid receptor antagonist	Oral	MDD (monotherapy or adjunctive)	Phase 2 trials ongoing; preclinical and early clinical data suggest promising anxiolytic and antidepressant effects	Investigational	–	Nausea, headache; long-term data limited

PPD, postpartum depression; NMDA, N-methyl-d-aspartate; RCTs, randomized controlled trials.

### *N*-methyl-d-aspartate and γ-aminobutyric acid receptor modulators

Glutamate and γ-aminobutyric acid (GABA) are the most abundant excitatory and inhibitory neurotransmitters in the brain, respectively. Accumulated evidence suggests that dysfunction of both glutamate and GABA or their balance may be a key factor for depression. Increased stress is associated with increased glutamate levels, which may be a factor for decreased synaptic connectivity (51). Neuroimaging studies suggest that there is a widespread GABA reduction in MDD brain, while alterations in glutamate are localized in brain regions such as the ventromedial prefrontal cortex (vmPFC) and the anterior cingulate cortex (ACC) (9). It is important to note that not all *N*-methyl-d-aspartate receptor (NMDA-R) antagonists, such as memantine and amantadine, are working antidepressants. This variability in efficacy may be attributed to the intricate nature of NMDA receptors. These receptors are heterotetrameric ion channels composed of a variety of subunits (GluN1, GluN2, and GluN3), each with numerous polymorphic variations. This complexity affords a vast array of potential binding sites, leading to a broad spectrum of clinical responses (53).

#### Ketamine and S-ketamine

After a long period of monoaminergic antidepressants, ketamine, a derivative of phencyclidine (PCP) is the first novel and rapid-acting agent. To our current knowledge, ketamine utilizes different pathways to treat depressive symptoms, as follows:

1. By non-competitively blocking NMDA-R on the inhibitor GABAergic neurons, decreasing the inhibitory effect of GABA on the pre-synaptic glutamate neurons, which allows more glutamate to act on AMPA receptors (AMPA-R) on post-synaptic glutamate neurons.
2. By increasing the synthesis of brain-derived neurotrophic factor (BDNF) in post-synaptic glutamatergic neurons. BDNF is acknowledged as an important peptide for synaptic neuroplasticity and is secreted retrogradely, activating mechanistic target of rapamycin complex 1 (mTORC1), a protein complex that functions as a nutrient/energy/redox sensor and controls protein synthesis.
3. By blocking the extracellular NMDA-R, which also increases mTORC1.
4. Some metabolites of ketamine, such as 2*R*,6*R*-NHK, directly potentiate the activation of AMPA-R.
5. Through reversal of the depression-associated changes in lateral habenula neuronal function (in animal models) (10).

The intravenous dosage is commonly 0.5 mg/kg, delivered for 30–40 min with blood pressure, heart rate, and temperature monitoring, and can be administered alone or as an adjunct to another antidepressant. Due to its possible side effects and its route of administration, many studies have focused on treatment-resistant depression (TRD). The results of seven placebo-controlled ketamine studies suggested a significant difference in outcome favoring ketamine, with a pooled odds ratio (OR) of 6.33 (95%CI = 3.33–12.05, *t* = 6.39, *p* < 0.0001) after 24 h of infusion (11). Ketamine appears to reduce depressive symptoms in a heterogeneous group of patients, showing even more benefits compared with placebo in those who have not responded to previous medication treatment (12). Currently, there is a lack of specific clinical or demographic features at the patient level that could guide ketamine treatment decisions. Similar efforts did not yield any clinically relevant results for blood-based biomarkers of antidepressant response to ketamine or S-ketamine (esketamine) (13).

Ketamine has two enantiomers: R-ketamine and S-ketamine. S-ketamine nasal spray was approved by the US Food and Drug Administration (FDA) in 2019 for TRD in conjugation with oral antidepressants and in 2020 for adults with MDD with acute suicidal ideation or behavior. Esketamine is administered twice weekly for the first month, then is decreased to once weekly. One of the large placebo-controlled trials (TRANFORM-2) for short-term treatment showed a trial advantage of ketamine over placebo; the other two (i.e., TRANFORM-1 and TRANFORM-3) could not separate from placebo, although TRANFORM-1 and TRANFORM-2 had similar decreases in the depression scores. TRANFORM-3 was on patients over 65 years old, with better treatment effects for younger patients (65–74 years) and patients with earlier onset of depression (<55 years) (14). In long-term relapse prevention studies (SUSTAIN-1 and SUSTAIN-2), patients who responded to esketamine+antidepressant showed a clear increase in the time to relapse compared with those on antidepressant+placebo esketamine (NNT = 4). When treatment was maintained for up to 6 months, relapse can be reduced by 51% in remitted patients if they are also taking their previous antidepressant (14, 15). When compared with the active comparator, esketamine was found superior to quetipine augmentation to antidepressants in both acute and maintenance treatments (16). In the KetECT study, when comparing ketamine infusions to electroconvulsive treatment (ECT), it was found that 46% of patients treated with ketamine infusions achieved remission. Conversely, among those undergoing ECT, a higher rate of remission was observed, with 63% of patients reaching this outcome (17).

In addition to these findings, real-world data further validated the effectiveness of esketamine. The REAL-ESK study (18) evaluated over 600 patients with TRD across multiple centers, demonstrating both safety and sustained antidepressant effects in clinical practice. Moreover, efforts have been made to identify predictors of response. For instance, Pettorruso et al. (19) explored a clinical algorithm incorporating demographic, symptom, and treatment history variables to personalize esketamine therapy. Rosso et al. (20) also presented data on the treatment trajectories of esketamine, identifying subgroups with early *vs*. delayed response patterns. Importantly, in a randomized trial comparing esketamine augmentation with quetiapine augmentation in TRD, esketamine showed superior efficacy in both acute and maintenance phases (16).

#### Dextromethorphan/bupropion

Dextromethorphan (DXM) is an NMDA-R antagonist and sigma-1 receptor agonist that has recently been approved for the treatment of MDD when combined with bupropion. Bupropion, beyond its role as a norepinephrine–dopamine reuptake inhibitor (NDRI), inhibits CYP2D6, the primary metabolic pathway for DXM, thereby enhancing its plasma levels and extending its therapeutic effect (21).

In a phase 2, double-blind, randomized controlled trial involving 80 participants, the combination of DXM and bupropion (45 mg/105 mg twice daily) demonstrated significantly greater reductions in the Montgomery–Asberg Depression Rating Scale (MantidepressantRS) scores compared with bupropion monotherapy by week 6. The remission rates reached 46.5% for the combination group *versus* 16.2% in the bupropion-only group (21).

These findings were corroborated in the larger phase 3 GEMINI trial, which enrolled 327 patients and found that 39.5% of those receiving the active combination achieved remission at week 6 compared with 17.3% in the placebo group. The response rates were 54.0% *versus* 34.0%, respectively (22). Adverse effects were mostly mild to moderate and included dizziness, dry mouth, decreased appetite, and anxiety. Based on this evidence, the FDA approved the DXM–bupropion combination for the treatment of MDD in 2022.

#### Esmethadone

Esmethadone (REL-1017) is the opioid-inactive enantiomer of methadone, designed to avoid the addictive properties of its counterpart, levomethadone. It exhibits minimal affinity for µ-opioid receptors, substantially lowering its abuse potential while retaining antidepressant activity through NMDA-R modulation.

In preclinical studies, esmethadone enhanced the synaptic protein expression in the medial prefrontal cortex and increased the BDNF levels, both of which are associated with antidepressant effects (23). These molecular changes support the hypothesis that esmethadone promotes synaptic plasticity and resilience to stress.

In a phase 1 clinical trial, a 10-day regimen of 25 mg esmethadone resulted in the plasma BDNF concentrations increasing up to 17-fold compared with the baseline. These changes were not observed in the placebo group, and a strong correlation between plasma BDNF and drug exposure was demonstrated (23).

Subsequently, a phase 2a, double-blind, randomized, placebo-controlled trial evaluated the efficacy of esmethadone over 7 days in patients with TRD who had failed to respond to one to three prior antidepressants. The study found significant improvements in depressive symptoms for both the 25- and 50-mg daily doses, with effect sizes of 0.9 and 1.0, respectively, sustained up to 7 days after treatment discontinuation (24).

Common side effects included headache, nausea, constipation, and somnolence, which were generally mild to moderate in severity. These promising early-phase results support further investigation of esmethadone as a fast-acting, non-opioid antidepressant option for TRD.

### Neurosteroids and γ-aminobutyric acid type A receptor modulators

#### Brexanolone

Brexanolone is an intravenous formulation of allopregnanolone, a neuroactive steroid and endogenous metabolite of progesterone. It functions as a positive allosteric modulator of GABA-A receptors, with affinity for both the synaptic and extrasynaptic receptor populations. This pharmacological profile distinguishes brexanolone from traditional benzodiazepines, which primarily target synaptic GABA-A subtypes and do not show equivalent antidepressant efficacy (25).

The levels of allopregnanolone increase dramatically during pregnancy and drop sharply after childbirth. These fluctuations are hypothesized to play a role in the development of postpartum depression (PPD) in biologically vulnerable individuals. Brexanolone is believed to stabilize the GABAergic neurotransmission during this critical transition, thus alleviating the mood symptoms associated with PPD.

In two large-scale, double-blind, randomized, placebo-controlled phase 3 trials, a 60-h continuous infusion of brexanolone resulted in rapid and significant improvements in the depression and anxiety scores compared with placebo, with effects lasting for at least 30 days (26). Notably, brexanolone was undetectable in maternal plasma 3 days after infusion, making it a potentially safe option for lactating patients.

The most commonly reported adverse effects included dizziness, somnolence, and headache. However, rare but serious risks such as excessive sedation, loss of consciousness, and hypoxia necessitate continuous inpatient monitoring during administration. Based on this clinical evidence, the FDA approved brexanolone in 2019 as the first medication specifically indicated for PPD.

#### Zuranolone

Zuranolone is a next-generation neuroactive steroid that acts as a positive allosteric modulator of GABA-A receptors, similar to brexanolone. However, zuranolone offers the major advantage of oral administration, allowing for outpatient use. Similarly to brexanolone, zuranolone targets both synaptic and extrasynaptic GABA-A receptors, potentially restoring the disrupted inhibitory tone associated with depression (50).

Initial trials have demonstrated the efficacy of zuranolone in PPD. In a randomized, placebo-controlled trial, zuranolone (30 or 50 mg daily for 14 days) led to a significant reduction in the Hamilton Depression Rating Scale (HAM-D) scores by day 15 compared with placebo (27). Additional studies confirmed its rapid-onset action, with improvements observed as early as day 3 (47, 48, 28).

Zuranolone has also been investigated in MDD. The phase 3 CORAL study assessed zuranolone co-initiated with standard antidepressants in adults with MDD. On day 3, significant improvements in depressive symptoms were observed in the zuranolone+antidepressant group compared with the placebo+antidepressant group, with an effect size of 0.38 (29). These findings suggest that zuranolone may enhance the early therapeutic response when used in combination with selective serotonin reuptake inhibitors (SSRIs) or serotonin–norepinephrine reuptake inhibitors (SNRIs).

A recent meta-analysis including seven randomized controlled trials concluded that zuranolone is a fast-acting and well-tolerated treatment for both PPD and MDD, with effects sustained up to 45 days after cessation (30). The most commonly reported adverse events were somnolence, dizziness, and sedation. Importantly, no significant withdrawal symptoms or safety concerns were observed, and the discontinuation rates did not differ from those of placebo.

The FDA approved zuranolone for PPD in 2023. Approval for MDD is pending further confirmatory data.

#### Ezogabine

Ezogabine (also known as retigabine) is a selective KCNQ2/3 potassium channel opener that was originally developed as an anticonvulsant. Recent preclinical and clinical evidence has suggested its potential as a novel antidepressant, particularly in addressing symptoms of anhedonia and motivational deficits associated with MDD.

In rodent models of chronic social defeat stress, a paradigm used to model depression, increased expression of KCNQ-type potassium channels in the ventral tegmental area (VTA) has been associated with stress resilience (49). The administration of ezogabine has been shown to reverse susceptibility to stress-induced depressive-like behaviors, likely through the modulation of dopaminergic neuron excitability in mesolimbic circuits (31, 32).

Building on these findings, early-phase clinical trials have explored the antidepressant effects of ezogabine in humans. In two small proof-of-concept randomized controlled studies, daily oral ezogabine significantly reduced the depressive symptoms and improved anhedonia, as measured by the MantidepressantRS and the Snaith–Hamilton Pleasure Scale (SHAPS), compared with placebo (33, 34).

Although these studies were limited by their small sample sizes and short durations, the results support the hypothesis that targeting neuronal excitability via KCNQ2/3 channels may represent a novel mechanism of action for the treatment of MDD. Further large-scale placebo-controlled trials are needed to fully evaluate the safety, efficacy, and tolerability of ezogabine in broader patient populations.

#### Navacaprant

Navacaprant (also known as CERC-501 or aticaprant) is a selective and potent antagonist of the κ-opioid receptor (KOR). KORs are activated by endogenous ligands such as dynorphins and are known to mediate dysphoria, anhedonia, and stress-induced depressive states. Antagonism of KORs has been proposed as a promising approach for alleviating the affective and motivational symptoms in MDD (56).

In a phase 2 randomized 8-week clinical trial involving 100 patients with moderate-to-severe MDD, oral navacaprant (80 mg/day) monotherapy significantly reduced the depressive symptoms, including anhedonia (54). These results support the mechanistic rationale for KOR antagonism as a therapeutic strategy in depression. Although detailed peer-reviewed publication of this study is pending, interim data suggest favorable tolerability and safety profiles.

Navacaprant has also been explored in comparison to other opioid modulators. For instance, the combination of buprenorphine and samidorphan—designed to leverage the KOR antagonism of buprenorphine while mitigating the effects of µ-opioids (57)—did not demonstrate sufficient efficacy in major depressive episodes and failed to receive FDA approval (35, 36). The shortcomings of buprenorphine-based strategies highlight the potential advantages of the selective KOR antagonism of navacaprant, without the complex receptor interactions associated with partial agonists or mixed activity.

Navacaprant is currently undergoing phase 3 trials, and if confirmed, its efficacy and oral formulation may position it as a valuable adjunctive or monotherapy option in TRD.

### Serotonergic psychedelics: psilocybin, DMT, LSD, and mescaline

There is increasing interest in utilizing psychedelic compounds to treat neuropsychiatric disorders, including depression (37). Previous research has indicated that the acute psychedelic effects of these substances are crucial to their positive outcomes, such as their antidepressant effects (38). These acute effects are believed to be connected to the sustained therapeutic benefits observed in patients over the long term. Psilocybin, dimethyltryptamine (DMT), lysergic acid diethylamide (LSD), and mescaline interact with various receptors in the brain, including 5-HT, dopamine, sigma, and trace amine-associated receptors (TAARs) (39). Serotonergic psychedelics, in particular, have a strong affinity for 5-HT receptors, particularly the 5-HT2A receptor. While various serotonergic psychedelics are being explored for their therapeutic potential, psilocybin is currently the most advanced in clinical research and is nearing phase 3 development (40).

Psilocybin, a naturally occurring serotonergic psychedelic compound, is currently among the most extensively studied psychedelic agents for the treatment of MDD and TRD. It primarily acts as a 5-HT2A receptor agonist, but also interacts with other 5-HT, dopamine, and TAARs, contributing to its complex psychopharmacological effects (39).

The therapeutic efficacy of psilocybin is believed to stem not only from its receptor-level activity but also from its capacity to induce profound acute psychological experiences, which may facilitate neuroplasticity, emotional processing, and therapeutic insight (38). These acute effects have been consistently associated with sustained antidepressant benefits in both open-label and placebo-controlled studies.

In a large phase 2 randomized clinical trial, Goodwin et al. (41) compared single doses of psilocybin (1, 10, and 25 mg) in TRD. The 25-mg group demonstrated significantly greater reductions in the MantidepressantRS scores compared with the 1-mg control. Similarly, Raison et al. (42) found that psilocybin significantly outperformed niacin as a placebo control in reducing depressive symptoms in patients with MDD.

Another pivotal study compared psilocybin to the SSRI escitalopram. In this head-to-head trial, psilocybin produced similar or superior antidepressant effects, with a faster onset and fewer side effects (43). Additional research has confirmed the durability of the antidepressant effects of psilocybin, with significant improvements persisting for several weeks following a single dose (44).

Despite promising data, psilocybin has not yet received regulatory approval and remains classified as a Schedule I substance in many jurisdictions. Ongoing phase 3 trials and evolving regulatory frameworks will determine its future role in clinical practice.

## Conclusion

The landscape of pharmacologic treatment for MDD is rapidly evolving. In contrast to conventional monoaminergic antidepressants, many of which require weeks to exert their therapeutic effects and fail to produce remission in a significant proportion of patients, the emerging agents reviewed here offer novel mechanisms, faster onset of action, and the potential to address specific symptom dimensions such as anhedonia, suicidality, and PPD (45, 46).

Agents such as esketamine and brexanolone have already received FDA approval, while zuranolone, esmethadone, and psilocybin are progressing through phase 3 trials with encouraging results. Others, such as ezogabine and navacaprant, have demonstrated potential in targeting previously underexplored neurobiological systems (29, 34, 41).

However, several barriers limit the routine implementation of these treatments in clinical practice. Firstly, long-term safety data remain limited for many agents, particularly psychedelics and esmethadone. Secondly, regulatory restrictions and public perception—especially concerning psychedelic compounds—continue to impede access. Thirdly, logistical challenges such as inpatient administration (e.g., 60-h infusion for brexanolone or IV ketamine) reduce scalability and add to healthcare costs (16, 26).

Furthermore, disparities in insurance coverage, cost-effectiveness, and clinician familiarity may hinder adoption. Future research should prioritize comparative effectiveness studies, optimal patient selection, response durability, and health economic assessments.

Overall, the field is witnessing a paradigm shift from traditional neurotransmitter-based treatments to mechanism-driven, circuit-level interventions, with a growing emphasis on neuroplasticity, stress resilience, and real-world applicability. If ongoing trials validate their efficacy and safety, these novel agents could redefine the standard of care for MDD in the coming decade.
